# G protein-coupled receptors and obesity

**DOI:** 10.3389/fendo.2023.1301017

**Published:** 2023-12-14

**Authors:** Alessandro Pocai

**Affiliations:** Cardiovascular and Metabolic Disease, Johnson & Johnson Innovative Medicine Research & Development, Spring House, PA, United States

**Keywords:** GLP-1, GIP, semaglutide, tirzepatide, glucagon, amylin, obesity

## Abstract

G protein-coupled receptors (GPCRs) have emerged as important drug targets for various chronic diseases, including obesity and diabetes. Obesity is a complex chronic disease that requires long term management predisposing to type 2 diabetes, heart disease, and some cancers. The therapeutic landscape for GPCR as targets of anti-obesity medications has undergone significant changes with the approval of semaglutide, the first peptide glucagon like peptide 1 receptor agonist (GLP-1RA) achieving double digit weight loss (≥10%) and cardiovascular benefits. The enhanced weight loss, with the expected beneficial effect on obesity-related complications and reduction of major adverse cardiovascular events (MACE), has propelled the commercial opportunity for the obesity market leading to new players entering the space. Significant progress has been made on approaches targeting GPCRs such as single peptides that simultaneously activate GIP and/or GCGR in addition to GLP1, oral tablet formulation of GLP-1, small molecules nonpeptidic oral GLP1R and fixed-dose combination as well as add-on therapy for patients already treated with a GLP-1 agonist.

## Introduction

G protein-coupled receptors are the largest family of membrane proteins targeted by approved drugs (1). Ligand binding results in activation of downstream pathways including G-proteins, β-arrestins and other non-G-protein transducers. Molecules that act by directly binding GPCRs are either orthosteric (bind in the same pocket as the natural ligand) or allosteric (bind in a different pocket). Allosteric modulators cooperatively interact with the natural orthosteric ligand to potentiate or attenuate receptor signaling. β-arrestins contribute to decreases in the response of the receptor to the respective agonist by internalization of the receptors. Activated GPCRs can also promote the activation of a subset of signaling pathways resulting in ‘ligand bias’ with unique cellular responses. Drugs targeting GPCRs such as GLP-1RA, glucose-dependent insulinotropic polypeptide receptor (GIPR), and melanocortin-4 receptors (MC4R) have been approved for weight management and T2D (2, 3).

Obesity is a chronic multifactorial disease (4) increasing the risk of heart disease, diabetes, liver disease, sleep apnea and certain cancers (5). It is estimated that by 2030 nearly 1 in 2 adults will be obese in US (6). In the past 5 years, major therapeutic advances have been made with multiple mechanisms progressing to the clinic and the approval of semaglutide, the first GLP-1RA leading to over 10% weight loss, an amount known to improve many of the complications associated with obesity (7). Recently semaglutide demonstrated also cardiovascular benefits (8). The increased efficacy has reduced the gap with the ~25-30% weight loss achieved with bariatric surgery during the first 2 years (9–12) and has driven additional efforts for a weight management market projected to reach 77 billion by 2030 (Weight Loss Drugs Boost Obesity Market Value | Morgan Stanley). Novel approaches have made significant progress such as oral tablet formulation of GLP-1, oral small molecules nonpeptidic GLP1R agonists and peptides dual or tri-agonists. Additional mechanisms are being developed such as PYY, GIP, amylin as a potential second activity to be combined with GLP-1RAs.

## Glucagon-like peptide-1 receptor (GLP-1R)

**Figure 1:**
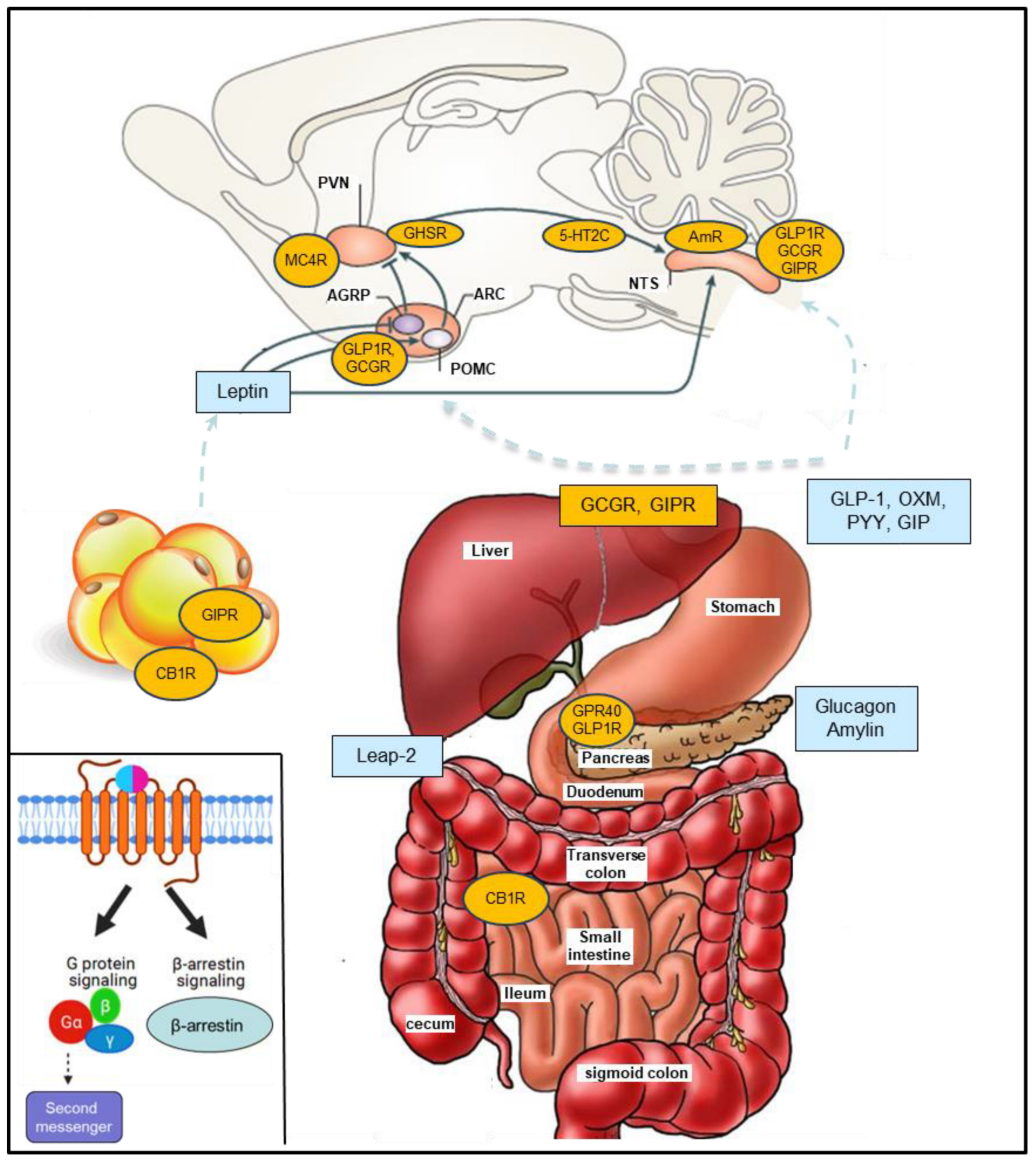
G-protein coupled receptors (GPCRs) and peptides discussed. The anorexigenic hormones (blue) are released from various organs mostly in the gastrointestinal system and pancreas and modulate GPCRs leading to food intake suppression, reduced gastric emptying and increased energy expenditure. Inset. GPCR signaling: Balanced agonists activate both the G protein- and β-arrestin-dependent signaling pathway while biased agonists selectively activate the GPCR-dependent signaling pathway affecting cellular response through second messenger activation or the β-arrestin-dependent signaling pathway. Glucagon-like peptide-1 (GLP-1), oxyntomodulin (OXM), gastric inhibitory polypeptide (GIP), peptide YY (PYY), cannabinoid receptor 1 (CB1R), Leap-2 (Liver-expressed antimicrobial peptide 2), melanocortin 4 receptor (MC4R), amylin receptor (AmR), 5-hydroxytryptamine receptor 2C (5-HT2CR). AgRP (Agouti-Related Protein), POMC (proopiomelanocortin); NTS, nucleus tractus solitarius; ARC, Arcuate nucleus; PVN, paraventricular nucleus.

Processing of the preproglucagon precursor by prohormone convertase 1/3 leads to production of GLP-1, GLP-2, oxyntomodulin, glucagon and glicentin in a tissue-specific manner (13–16). GLP-1 is a peptide hormone mainly secreted from the gut L cells that improve glucose homeostasis and promote weight loss (**Figure 1**) (13, 15–19). Liraglutide (Saxenda) and semaglutide (Wegovy), the first and the second GLP-1RA approved for weight management, achieved mean weight change from baseline of -6.4% vs -15.8% respectively in a head-to-head trial (20–22). In a recent landmark trial (SELECT trial), in over 17,000 overweight or obese non-diabetic adults with established cardiovascular disease, semaglutide reduced major adverse cardiovascular events by 20%, compared to placebo (8, 23).

## Oral GLP-1

Although the injectable treatments have reached double digit weight loss, oral therapies are more cost effective and are more likely to achieve long term efficacy through better compliance and combination strategies (24). An oral formulation of semaglutide (14 mg) with the permeation enhancer salcaprozate sodium (SNAC) (25) has been approved as first-line therapy in adults with T2D (Rybelsus) and recent data suggest that 50 mg oral semaglutide results in an efficacy profile comparable to injectable semaglutide (26). However, to achieve sufficient absorption, Rybelsus requires food and water restrictions, an absorption enhancer and is less cost effective due to approximately 1% absorption (27). Two oral non peptidics small molecule GLP-1RA have completed phase 2 trials for weight management and T2D: Once-daily Orforglipron (LY3502970) and twice daily Danuglipron (PF-06882961). Orforglipron, a biased partial agonist of the GLP-1R with lower activation of the β-arrestin pathway (28), resulted in body weight reductions up to 12.6% (placebo adjusted) at 36 weeks in obese non-diabetic patients (29) while Danuglipron, a GLP-1R full agonist achieved approximately up to 4-5% body weight lowering at 16 weeks in T2D (30, 31).

## Glucagon receptor (GCGR)

GCGR is a family B GPCR activated by glucagon mainly expressed in liver and kidney and, to a lower extent in heart, adipose tissue, pancreas, gastrointestinal tract and other tissues. Glucagon suppresses food intake and stimulates energy expenditure and hepatic glucose production (32–34). Consistently, administration of GCGR antagonists in patients with T2D resulted in decreased glucose levels (35) but also increased body weight providing human support for glucagon receptor agonism (36). Moreover, oxyntomodulin, a gut peptide co-secreted with GLP-1 with promising weight-loss and glucose-lowering properties in humans (37, 38), is a dual agonist at the GLP-1R and GCGR (39–41). Because GLP-1 improves body weight and glucose metabolism in humans (42, 43), designing single peptides co-agonists at GLP-1R and GCGR may produce superior body weight benefits to GLP-1R agonism alone and could mitigate the effect of glucagon on glucose production. Two independent papers reported for the first time the use of GLP1R/GCGR dual agonists as being of enhanced efficacy relative to pure GLP1R agonists in the treatment of rodent obesity, with simultaneous improvement in glycemic control (44, 45). Importantly, rodent models suggest that receptor balance is critical to optimize efficacy and safety (46). Translational superiority of GLP-1R/GCGR dual agonism versus GLP-1R on the body weight benefit was later demonstrated in obese rhesus monkeys (47, 48) and confirmed by other groups (49). Recently, Survodutide (BI 456906), a once weekly GLP1/GCGR dual agonist, showed up to 19% weight loss after 46 weeks in overweight and obese subjects (50). Other companies have active efforts in this space such as efinopegdutide (MK-6024) (51), pemvidutide (Altimmune, ALT-801) and mazdutide (LY3305677, IBI362, 52).

## Glucose-dependent insulinotropic polypeptide receptor (GIPR)

GIP is an incretin hormone secreted from K cells in the proximal small intestine that promote nutrient-stimulated insulin release through GIPR (53–56). GIPR activation stimulates glucagon secretion, lipid accumulation in adipose tissue, bone formation and modulation of food intake and preference (57–61). Genetic and pharmacological inhibition of GIP and GIPR in mice protects from obesity (62–64). These data supported GIPR antagonism for obesity while GIP receptor agonism was thought to be ineffective for glucose lowering in T2D patients and potentially deleterious for body weight (65, 66). However, following the demonstration of superior pharmacology of GLP-1R and Glucagon receptor co-agonism to GLP-1RA (44, 45), it was discovered that also GIPR agonism can bring synergistic efficacy when combined with GLP-1 agonism (67–70). This effort resulted in the approval of Tirzepatide for T2D (Mounjaro) and obesity (Zepbound) (71–74; www.fda.gov/news-events/press-announcements/fda-approves-new-medication-chronic-weight-management). Tirzepatide favors GIPR over GLP-1R activity and it is biased for cAMP over β-arrestin recruitment at GLP-1R (74–76).

Following the initial reports of pharmacological and genetic deletion of GIPR, Killion et al. showed that an antagonist mAb to the GIPR resulted in food intake and body weight reduction in obese non-human primates and that the combination of GLP-1 agonist and GIPR antagonism resulted in additive weight loss (68, 77). This directionality was also supported by rare variants in GIPR that contribute independently to a lower body weight (78). This approach is being explored by Amgen with Maridebart cafraglutide (formerly AMG 133) which is formed by two GLP-1 agonist peptides conjugated to an anti-GIP antibody backbone (79). Killion recently reported that chronic GIP agonism desensitizes and inhibits the activity of GIPR at least in adipose tissue potentially reconciling the similar body weight directionality observed with agonists and antagonists (69).

## GLP1R/GIPR/GCGR – TRI-agonists

The advancement of dual agonists for GLP-1/GIP and GLP-1/GCGR has also reinvigorated the development of triagonist peptides at the GLP-1R, GIP and GCGR (80–82). The results of a phase 2 trial of the triple agonist LY3437943 (Retatrutide) showed 24.2% weight loss at the highest dose (12 mg) in 48 weeks (23, 83). Eli Lilly recently announced initiation of 3 large phase 3 studies (TRIUMPH) of retatrutide. Multiple triple-agonists are in development including HM15211, NN9423, SAR441255.

## Amylin receptor

The amylin receptor (AmR) is a GPCR composed of the calcitonin receptor (CTR) complexed with different receptor -activity -modifying proteins (RAMP) 1 or RAMP3. Amylin is a neuropeptide co-secreted with insulin by pancreatic beta-cells which slows gastric emptying, and decreases food intake (84, 85). Pramlintide is an injectable amylin analogue dosed three times a day as an adjunct for the management of T1D and T2D resulting in modest weight loss, smaller meal size and less binge eating episodes (86). CagriSema is a once-weekly injection of semaglutide and the long-acting amylin analogue cagrilintide (AM833, 87, 88) leading to nearly 16% reduction in body weight (89, 90). Recently Novo Nordisk announced the initiation of REDEFINE 4, a phase 3 trial comparing CagriSema 2.4 mg with Lilly’s Zepbound 15 mg evaluating weight loss after 72 weeks in 800 obese patients non-diabetic and is expected to be completed by October 2025 (NCT06131437). Amycretin (NNC0487-0111) is an oral formulated GLP-1 and amylin peptide being tested in phase 1 (NCT06049329). Other approaches include GUC17 (Gubra), ZP8396 (91–93), KBP-066A (94).

## Neuropeptide Y receptor type 2 (Y2R)

Peptide YY (PYY) is secreted post-prandially by the L-cells of the gut together with GLP-1 (95). Secreted as PYY(1–36), it is then cleaved by the enzyme dipeptidyl peptidase 4 into PYY(3–36). PYY(3–36) activates Y2 receptors in the hypothalamus leading to appetite reduction and food reward regulation (96, 97). Bariatric surgery results in increased secretion of GLP-1 and PYY and co-infusion of PYY3–36 and GLP-1 elicit at least additive anorectic effect with ~30% reduction in food intake exceeding the 15%–20% reduction predicted to sustain a weight loss ≥10% in one year (98–101). PYY-based therapeutic development remains challenging due to emesis and low stability (102). Recent progress includes an antibody NPY2R agonist with infusion-like exposure resulting in reduction of food intake without emesis (101), Y14, a zinc-based extended-release selective Y2 receptor agonist, that demonstrated preliminary efficacy and tolerability in human subjects (103), and BI 1820237, a long acting NPY2 receptor agonist that showed decreased energy intake and delayed gastric emptying in overweight or obese men (104). Additional companies active in this space include Carmot Therapeutics and Cinfina Pharma.

## Melanocortin 4 receptor (MC4R)

MC4R have been involved in food intake, metabolism, sexual behavior, and male erectile function (105, 106). Small molecules and peptide agonists of MC4R have been evaluated for obesity including MK-0493, LY2112688, MC4-NN-0453, PF-00446687 and AZD2820 and failed due to HR and BP changes, lack of human efficacy or tolerability (106). Setmelanotide (Imcivree) received FDA approval in 2020 for chronic weight management in obesity caused by genetic defects and did not result in obvious undesirable cardiovascular effects. In 2022 setmelanotide was also approved for patients with Bardet-Biedl Syndrome. Receptor selectivity, differential brain penetration and biased signaling may explain the differential profile (107, 108). Protective MC4R variants identified in the UK Biobank exhibited signaling bias toward β-arrestin recruitment and increased MAPK pathway activation suggesting that β-arrestin-biased MC4R signaling may represent an effective strategy for weight loss (109, 110). Setmelanotide has a higher potency for cAMP and a weaker effect on ERK1/2 phosphorylation when compared to alpha-MSH indicating biased agonism (110, 111).

## Cannabinoid receptor 1 (CB1R)

The CB1R is a G-protein coupled receptor highly expressed in the central nervous system. CB1R antagonist/inverse agonist, rimonabant, was approved for the treatment of obesity but it was withdrawn from the market due to neuropsychiatric adverse effects (112). Multiple strategies have been proposed to circumvent the neuropsychiatric adverse effects including biased signaling, allosteric modulators, neutral antagonists, and peripherally restricted ligands since several metabolic processes appear to benefit from peripheral blockade of CB1 (113–115). Recently Novo Nordisk announced the acquisition of INV-202, an oral CB1 inverse agonist designed to preferentially block CB1R in peripheral tissues. INV-202 administration for 28 days to adult subjects resulted in an average weight loss of 3.3% (vs 0.5% gain for placebo) (https://www.novonordisk.com/news-and-media/news-and-ir-materials/news-details.html?id=166304). Zizzari demonstrated that CB1 and GLP-1 receptors modulate food intake and body weight via reciprocal functional interactions achieving greater reduction in body weight than each individual monotherapy (116).

## Other GPCR targeted for the regulation of energy homeostasis

### 5-hydroxytryptamine receptor 2C (5-HT2CR)

Rodent studies have demonstrated that 5-HT2CR mediates most of the food intake and body weight effects of d-fenfluramine and sibutramine, but it was withdrawn from clinical use due to increased heart rate and blood pressure (117). Lorcaserin is a 5-HT2CR agonist approved for weight management, but it was withdrawn in 2020 due to a numerical imbalance in the cancers occurring in an over 12,000-participant clinical trial (118). Since then, progress has been made in 5-HT2CR research with the identification of allosteric modulators (119) acting on specific site of the receptor (120). Recently, Wagner et al. demonstrated that PPG neurons in the brainstem mediate the reduction of food intake by lorcaserin and that the combination of lorcaserin with GLP-1RA resulted in additive effects on food intake potentially providing a strategy to increase the therapeutic margin of future 5-HT2CR agonists (121).

## Ghrelin receptor (GHSR)

Ghrelin administration stimulates feeding, whereas GHSR antagonists inhibit feeding (122–125). Considering the high constitutive activity of GHSR, antagonists or inverse agonists of the GHSR have been proposed as potential approaches (126). Since the GHSR system also modulates other physiological functions such as GH secretion, it is necessary to expand understanding of the pathways involved and develop biased ligands to modulate only the therapeutically relevant signaling pathway (127). LEAP-2 (Liver-expressed antimicrobial peptide 2) has been characterized as an endogenous competitive antagonist of ghrelin and proposed as a potential therapeutic target for obesity (128, 129). In a small randomized, double-blind, placebo-controlled, crossover trial, LEAP2 infusion reduced postprandial glucose excursions, growth hormone concentrations and ad libitum food intake in healthy men (130).

## G protein-coupled receptor 40 (GPR40)

The orthosteric partial GPR40 agonist TAK-875 (fasiglifam) resulted in glucose improvement in T2D patients (131) but the development was halted in Phase III due to potential liver toxicity (132). Recently, GPR40 AgoPAM agonists have been identified resulting in body weight lowering and improvement of glycemic control by stimulating the secretion of insulin, glucagon, and gut peptides (133–136). Following the initial GPR40 AgoPAMs triggering rebound hyperglycemia at high doses, compounds were discovered with improved aqueous solubility that did not result in this side effect (137).

## Weight loss and fat free mass

One concern among new generation drugs is the rapid weight loss and its impact on muscle mass. Sarcopenia is common among older adults with obesity and NASH patients with cirrhosis leading to increased risk for lower bone density, decreased strength and mortality. A subgroup of participants treated with Tirzepatide underwent DEXA at baseline and at week 72 in the SURMOUNT-1 trial. Tirzepatide treatment resulted in -33.9% reduction in total body fat mass (vs -8.2% placebo) and reduced lean mass by -10.9% (vs -2.6% placebo). While the percent reduction in fat mass was approximately three times greater than the reduction in lean mass, the reduction in lean mass reported is typically observed over a decade or more in elderly patients (138). Moreover, dual and triple agonists such as Retatrutide lead to chronic glucagon receptor activation that can theoretically contribute to whole-body protein catabolism (139–141). Recently Lilly announced acquisition of Versanis’ lead obesity candidate bimagrumab, a monoclonal antibody targeting activin type II receptors to inhibit atrophy and to increase muscle mass (142–144). In a 48-week phase 2 trial, intravenous bimagrumab conferred approximately a 20% reduction in fat mass and a 4.4% increase in lean mass (143). A phase 2 study is ongoing to assess if bimagrumab in addition to semaglutide is able to preserve/increase muscle mass (NCT05616013).

## Discussion

**Figure 2:**
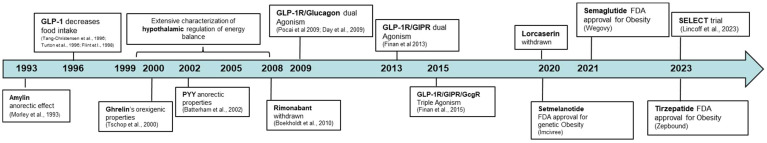
Timeline and discoveries discussed in this minireview with therapeutic relevance in obesity.

The therapeutic landscape for anti-obesity medications has drastically changed in the last few years with the approval of treatments for monogenic forms of obesity and a new generation of anti-obesity medications (**Figure 2**) (2, 108). These new anti-obesity medications are associated with over 10% body weight loss known to improve many of the complications associated with obesity. The published data on tirzepatide suggest that second- and third-generation dual agonists and tritagonists, nicknamed “double G” or “triple G”, have the potential to be superior to semaglutide in obese patients (71, 145, 146). A head-to-head trial of tirzepatide vs. semaglutide in adults with overweight or obesity (SURMOUNT-5) was initiated in April 2023 and is expected to be completed in November 2024 (NCT05822830). Thanks to major advancements on GPCR signaling in regulating energy homeostasis, we have a better understanding of the multi-state model where the ligands’ binding affects specific downstream pathways resulting in efforts targeting selective signaling pathways (biased signaling). Interestingly, biased agonism at the GLP-1R favoring cAMP generation over β-arrestin recruitment could be involved in the efficacy of tirzepatide resulting in less receptor internalization. The clinical success of tirzepatide has revamped the attention toward GLP-1R/GCGR dual agonists and tri-agonists GLP-1R/GCGR/GIPR in which the identification of an ideal receptor balance is required to maximize efficacy and mitigate safety concerns. An important consideration for dual and tri-agonist peptides, is to monitor and clarify the clinical consequences on the cardiovascular system, especially the heart rate increase (147, 148). GLP-1R agonism causes a small rise (usually 2–3 bpm) in heart rate (149), GIP infusion increases heart rate in humans (60) and GCGR agonism appears to have a positive chronotropic and inotropic action on the heart (150). Clinical data so far have shown that retatrutide dose-dependently increases heart rate up to 6.7 bpm at 24 weeks and declined thereafter (83) while pulse rate increased with increasing doses of tirzepatide peaking (6.6. bpm) at 20 weeks and then declining (151).

Importantly, in the SELECT trial, differences in rates of cardiovascular events between semaglutide and placebo began to emerge within the first few months suggesting that weight loss alone may not account for the full benefits (8). SURMOUNT-MMO, an event-driven cardiovascular efficacy trial evaluating tirzepatide in adults with obesity, is expected to be completed in October 2027 (NCT05556512).

On the co-morbidities front, GLP-1R is not expressed in the liver and the data generated with semaglutide in NASH patients seems to support a lack of a direct antifibrotic effect (152). Hence, GLP-1 therapeutics that target glucagon (and GIP) may have advantages over selective GLP-1RA for liver diseases. Recently, the kidney outcome trial FLOW evaluating semaglutide in T2D and chronic kidney disease (NCT03819153; 153), was stopped early for efficacy and it is expected to read out during the first half of 2024 (154).

There is also tremendous activity in the development of non peptidic small molecules targeting the incretin system to produce drugs that are orally available. Some oral GLP-1R agonists have achieved impressive weight loss results in phase 2 but appears to have a higher rate of discontinuation due to nausea and vomiting despite an onerous titration. As with all systemic small molecules there is also the potential for off-target toxicities and drug-drug interactions that need to be evaluated. In addition to confirm comparable efficacy to injectables, oral small molecules need to show the beneficial cardiovascular effects seen with semaglutide. In the future it will be interesting to see whether a single small molecule can target multiple family B GPCR receptors. Major progress has been made in the GLP-1 combination front with long-acting amylin analogues and a better understanding of the tolerability events that have limited development of PYY therapeutics. Interestingly, GIPR agonism blocks PYY (155) and GLP-1 receptor mediated emesis and illness behaviors in preclinical species (156). Addressing the translatability and the mechanism(s) involved in the anti-emetic actions of GIP, may lead to next generation therapeutics without the onerous titration and the tolerability issues of current therapeutics.

Despite the impressive results achieved, weight reduction is still considerably lower than bariatric surgery leaving important unmet medical needs and opportunities for obesity treatments. Differences in receptor balance and signaling, tissue distribution and penetration together with a greater understanding of pathways involved in body weight regulation and tolerability leave vast opportunities available to improve upon current therapeutics.

## Author contributions

AP: Writing – original draft, Writing – review & editing.
